# Expression of Bmi-1 in Pediatric Brain Tumors as a New Independent Prognostic Marker of Patient Survival

**DOI:** 10.1155/2013/192548

**Published:** 2013-07-28

**Authors:** Shirin Farivar, Reza Zati Keikha, Reza Shiari, Farzaneh Jadali

**Affiliations:** ^1^Department of Genetics, Faculty of Biological Science, Shahid Beheshti University (GC), Tehran 1983963113, Iran; ^2^Laser and Plasma Research Institute, Shahid Beheshti University (GC), Tehran 1983963113, Iran; ^3^Department of Pediatrics, Shahid Beheshti University of Medical Sciences, Mofid Children's Hospital, Tehran, Iran; ^4^Pediatrics Infectious Research Center (PIRC), Shahid Beheshti University of Medical Sciences, Tehran, Iran

## Abstract

*Objectives*. The B-cell-specific moloney leukemia virus insertion site 1 (the Bmi-1) gene is an important member in the family of polycomb group (PcG) genes that plays an oncogenic role in several types of cancer, but it's expression as a prognostic marker in pediatric brain tumors has not been indicated. *Materials and Methods*. The Bmi-1 gene expression, clinic pathological and prognostic significance in a series of pediatric brain tumors were examined by real-time PCR method in 56 pediatric brain tumors. *Results*. The Bmi-1 gene expression in various types of pediatric brain tumors compared to that in normal brain tissue was 4.85-fold. The relative expression varied from 8.64-fold in ependymomas to 2.89-fold in other types. Expression level in high-grade tumors compared to that in low-grade tumors was 2.5 times. In univariate survival analysis of the pediatric brain tumors, a significant association of high expression of the Bmi-1 with patient survival was demonstrated. In multivariate analysis, the Bmi-1 high expression provided significant independent prognostic factors. *Conclusion*. Increased expression of the Bmi-1 in pediatric brain tumors may be important in the acquisition of an aggressive phenotype. In addition, it can be used as a strong and independent molecular marker of prognosis in pediatric brain tumors.

## 1. Introduction

Brain tumors are the most common solid tumors and the second most frequent malignancy after leukemia in children [1]. So far, little is known about molecular mechanisms associated with these types of tumors. These tumors are the genotypically and phenotypically different from adult brain tumors [2, 3]. Five-year survival rate for children with brain tumors is estimated to be approximately 75% [4]. Gliomas comprises approximately 60% of cases among pediatric brain tumors, whereas the remaining 40% are heterogeneous and consist of medulloblastomas and other embryonal tumors (26%), craniopharyngiomas (4%), pineal tumors (1%), meningiomas (1%), and others [5]. Identification of molecular markers associated with pediatric brain tumors provides suitable conditions for treatment of these tumors. The B-cell-specific moloney leukemia virus insertion site 1 (Bmi-1) gene is an important member of the PRC1 complex. Members of PRC1 complex have been identified as transcriptional repressors [6–8]. The Bmi-1 mediates gene silencing by regulating chromatin structure, and it is essential for the maintenance and self-renewal of both haematopoietic and neural stem cells [9, 10]. This function of Bmi-1 depends on its ability to repress the INK4A locus. The INK4A locus as a target of Bmi-1 is a critical regulator of the p53 and Rb tumor suppressor pathways [11]. Thus, Bmi-1 would be the determining factor in the control of cell cycle. Overexpression of the Bmi-1 was found in high-grade B-cell non-Hodgkin Lymphomas (NHLs) [12], colorectal cancers [13], ovarian cancer, breast, cervical [14], skin cancer [15], neuroblastoma [16], and nonsmall cell lung cancer (NSCLC) [11]. Bmi-1 is known to be a useful prognostic marker in many cancers, including nasopharyngeal carcinoma [17], bladder cancer [18], gastric cancer [19], and others. The Bmi-1 expression as a prognostic marker in pediatric brain tumors has not been indicated. In this study, the Bmi-1 gene expression, clinicopathological, and prognostic significance in a series of pediatric brain tumors were examined.

## 2. Materials and Methods

### 2.1. Patients

A total of 56 pediatric brain tumors were obtained from archives of paraffin-embedded tissues between 2006 and 2012 at the Department of Pathology in Mofid Children's Hospital, Shahid Beheshti University of Medical Sciences. The selection of patients was based on distinctive pathologic diagnosis, availability of tissue, follow-up data, and not receiving radiation or chemotherapy. Pathological parameters and patient survival data were collected. These patients included 53.5% male and 46.5% female with mean age of 3.28 years. The pediatric brain tumor cases encompassed 20% medulloblastoma, 34% astrocytomas, 28% ependymomas, and 18% others (Primitive Neuro Ectodermal Tumors (PNET), gangliogliomas, and oligodendrogliomas). Clinicopathological characteristics of these patients were detailed in Table 1.

### 2.2. RNA Extraction and cDNA Synthesis

This protocol was used for extraction of total RNA from FFPE (formalin-fixed paraffin-embedded tissues) specimens [20]. Tissue sections were deparaffinized by 1.5 mL xylene at 37°C for 20 min and incubated in 1.5 mL 100% ethanol at 37°C for 30 min. 600 *μ*L of RNA lysis buffer containing 10 mmol/L TrisHcl (pH8), 0.1 mmol/L EDTA (pH8), and 2% SDS (pH7.3) with 50 *μ*L of 60 mg/mL proteinase K (Fermentas, Vilnius, and Lithuania) is added and incubated at 60°C for 16–20 h. Then total RNA was extracted by phenol chloroform method. The RNA concentration was measured using the Nano Drop 2000 (Thermo scientific, USA). Total RNA treated with DNase I (Invitrogen, Paisley, UK) to eliminate genomic DNA, for cDNA synthesis cDNA Accu Power Cycle Script RT PreMix (BioneerCo. Chungwon, South Korea), was used according to the manufacturer's instructions.

### 2.3. Real-Time PCR

Specific primers were designed for the Bmi-1 and Beta-actin as an internal control (Gen Bank accession numbers: NM-005180.8, NM-001101.3, Bmi-1 Forward: CTGCAGCTCGCTTCAAGATG. Bmi-1 Reverse: CACACACATCAGGTGGGGAT. Beta-actin Forward: ATGACTTAGTTGCGTTACACC. Beta-actin Reverse: TGCTGTCACCTTCACCGTTC) using oligo 7 software. 

Real-time PCR reactions were performed using SYBR Green method with Accu Power Green Star qPCR Master Mix (BioneerCo. Chungwon, South Korea) on a Rotor-Gene 6000 (Corbett Research, Sydney, Australia). PCR was run as follows: 94°C for 10 min, amplification for 40 cycles with denaturation at 94°C for 10 seconds, annealing at 57°C for 10 seconds, and extension at 72°C for 15 seconds. All experiments were repeated in duplicate or triplicate. Relative expression results of real-time PCR were carried out by REST 2009 software (Relative Expression Software Tool, Corbett Research).

### 2.4. Statistical Analysis

Statistical analysis was implemented by using the SPSS statistical software package (standard version 18.0; SPSS, Chicago, USA). The association of the Bmi-1 gene expression with clinicopathological characteristics of pediatric brain tumor patients was assessed by *T* test and ANOVA test. The Kaplan Meier method was used for survival analysis, and a log-rank test was used to compare the differences among survival curves. The COX risk ratio model was used for multifactorial analysis on survival, where *P* < 0.05 had statistical significance.

## 3. Results

### 3.1. Expression of the Bmi-1 in Pediatric Brain Tumors

The mean gene expression level of the Bmi-1 is used to segregate the patients into a low Bmi-1 gene expression (Bmi-1 expression < mean; *n* = 32) and a high Bmi-1gene expression (Bmi-1 expression > mean; *n* = 24). The Bmi-1 gene expression in various types of pediatric brain tumors compared to normal brain tissue was 4.85-fold (*P* = 0.009). The relative expression varied from 8.64-fold in ependymomas to 2.89-fold in other types. Expression level in high-grade tumors compared to low-grade tumors was 2.5 times (6.85 versus 2.75-fold change). The Bmi-1 gene expression in embryonal and gliomas tumors was 3.59- and 5.54-fold, respectively. The *T* test showed a significant difference between the Bmi-1 gene expression, embryonal/gliomas tumor, and high-grade/low-grade tumor (*P* < 0.05, Table 1).

### 3.2. Relationship between Clinicopathological Characteristics, Bmi-1 Gene Expression, and Pediatric Brain Tumor Patient Survival

**Figure 1:**
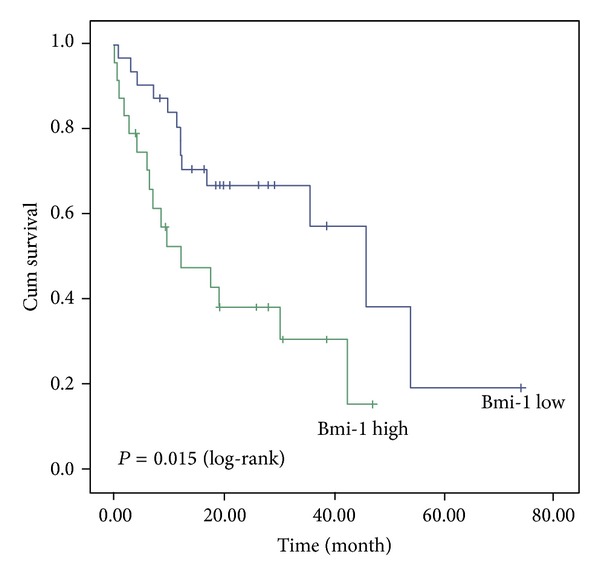
Univariate survival analysis of the Bmi-1 gene expression in all 56 pediatric brain tumors (32 patients with high expression and 24 patients with low expression). The Bmi-1 expression is a prognostic factor for survival (*P* = 0.015).

In univariate survival analysis, the cumulative survival Kaplan-Meier curves were calculated according to the test. Differences in survival time were examined by Log-rank test. Kaplan-Meier curves showed a significant influence of the Bmi-1 gene expression (*P* = 0.015), high-grade/low-grade (*P* = 0.011) on patient survival. The mean survival time for patient with high expression levels of the Bmi-1 was 20.3 months compared to 39.6 months for patients with low expression levels of the Bmi-1. The mean survival time was 24.6 months in high-grade tumors and 34.4 months in low-grade tumors. The time for M1, M2, M3, and M4 tumor stage was 37.6, 29.5, 24.9, and 20.1 months, respectively (Table 2). The comparison between survival curves of high expressions and low expression of the Bmi-1 is shown in Figure 1.

### 3.3. Independent Prognostic Factors of Pediatric Brain Tumors

#### 3.3.1. Multivariate Cox Regression Analysis

The Bmi-1 expression and other clinicopathological parameters that were significant in univariate analysis (high grade/low grade) were examined in multivariate analysis. The results of this analysis showed that the Bmi-1 gene expression (B = 0.827, standard error = 0.403, Wald = 4.207, relative rate = 2.287, and *P* < 0.05) and high grade/low grade (B = 0.751, standard error = 0.393, Wald = 3.647, relative rate = 2.118, and *P* < 0.05) were independent prognostic factors of a patient with pediatric brain tumor.

## 4. Discussion

Pediatric brain tumors are the most common solid tumors of childhood which approximately 1,500 patients suffer from every year in the United States. These tumors are second in order after leukemia in overall cancer incidences and responsible for a high proportion of deaths [21–23]. Considering the high incidence and mortality of patients with pediatric brain tumors identifying a genetic molecular marker that is associated with patient survival is necessary. The gene, the Bmi-1, is an important member in the family of polycomb group genes that participates in the regulation of cell growth and proliferation as transcriptional repressor [6–8]. It is essential for self-renewal of both hematopoietic, neural stem cells and cancer stem cells [9, 10, 24, 25]. The Bmi-1 proto-oncogene has been upregulated in a large number of cancers such as breast cancer [14], skin cancer [15], lymphoma [12], leukemia [26], and other cancers and is known to be a useful prognostic marker in many cancers. As it is so widely expressed, the Bmi-1 expression cannot be used as a specific marker for pediatric brain tumors. This is the first study that evaluates the Bmi-1 gene expression by real-time PCR in various types of pediatric brain tumors in association with clinicopathological and prognostic significance. The results demonstrated that the expression level of the Bmi-1 was high in all types of the pediatric brain tumors, suggesting that the Bmi-1 gene expression is related to the incidence of pediatric brain tumors. A significant increasing expression of the Bmi-1 was observed from high-grade to low-grade tumors. These findings show that upregulated expression of the Bmi-1 in high-grade tumors may acquire phenotypic characteristics of malignant tumor cells. It also shows that at high expression levels, tumors biological behavior gets worse. We found that the expression of the Bmi-1 in gliomas tumors was significantly larger than embryonal tumors. It suggests that the Bmi-1 gene plays some roles in the progression of gliomas tumors. Therefore, clinically, the measurement of the Bmi-1 gene expression could be helpful in diagnosis of gliomas from embryonal tumors. Moreover, we found that the Bmi-1 gene expression and high- or low-grade tumors were predictor of survival as evidenced by Kaplan-Meier curves and long rank test. Multivariate Cox proportional hazards regression analysis demonstrated that high expression of the Bmi-1 in pediatric brain tumors was a predictor of short overall survival, independent of high or low grade tumors. These findings show the important role of the Bmi-1 in pediatric brain tumors. Aberrant expression of the Bmi-1 in this tumors would change the composition of the PcG complex to proliferation cell cycle by transcription repressor of some genes such as p16Ink4A and p19Arf involved in tumor suppression, resulting in oncogenic effects, because the amounts of the Bmi-1 in the PcG complex determine its biochemical and biologic functions [27]. Moreover, overexpression of the Bmi-1 correlated with overexpression of PATCHED-1 (PTCH1), which is a reliable indicator of activation of the Shh pathway [27]. The abnormal activation of the Shh pathway is important in a subset of pediatric brain tumors [28]. Also it has been postulated that the Bmi-1 plays a role in self-renewal of cancer stem cells [24], so high expression of the Bmi-1 correlates with greater capacity of self-renewal and might represent the tumorigenesis and poor prognosis in pediatric brain tumors. We observed that data generated from FFPE material are comparable to data extracted from the fresh frozen samples; however, there was no significant association between the gene expression level and the time passed after formalin-fixed paraffin embedding.

## 5. Conclusion

In this study, to our knowledge, for the first time, we describe the Bmi-1 gene expression in various types of pediatric brain tumors. Our results demonstrated that increased expression of the Bmi-1 in pediatric brain tumors may be important in the acquisition of an aggressive phenotype. In addition, our study introduces the Bmi-1 expression as a strong and independent molecular marker of prognosis in pediatric brain tumors in addition to providing a specific target site for targeting therapy.

## Figures and Tables

**Table 1:** Correlation of the Bmi-1 gene expression to clinico-pathologic features of pediatric brain tumors.

	Cases	The Bmi-1 gene expression (fold change)	*P* value
All tumors	56	4.85	**0.009**
Sex			0.75
Male	30	4.50	
Female	26	5.35	
Embryonal/gliomas			**0.046**
Embryonal	16	3.59	
Gliomas	40	5.54	
Tumor grade			**0.03**
High grade	26	6.85	
Low grade	30	2.75	
Tumor type			0.061
Medulloblastoma	11	3.53	
Astrocytomas	19	5.11	
Ependymomas	16	8.64	
Other	10	2.89	

**Table 2:** Univariate survival analysis (Kaplan-Meier): survival times according to clinicopathological features and the Bmi-1 gene expression.

	Case	Mean survival time (months ± s.e.)	Chi-square	*P* value
Bmi-1 expression			5.87	**0.015**
High expression	24	20.31 ± 3.84		
Low expression	32	39.65 ± 5.90		
Sex			0.038	0.845
Male	30	35.28 ± 7.43		
Female	26	28.65 ± 4.25		
Embryonal/gliomas			1.88	0.17
Embryonal	16	36.23 ± 6.06		
Gliomas	40	23.53 ± 4.01		
Tumor grade			6.41	**0.011**
High grade	26	24.62 ± 5.52		
Low grade	30	34.44 ± 3.23		
Tumor type			1.43	0.21
Medulloblastoma	11	28.41 ± 4.47		
Astrocytomas	19	27.76 ± 5.44		
Ependymomas	16	19.46 ± 4.48		
Other	10	31.38 ± 7.77		
